# *In‐situ* 2D bacterial crystal growth as a function of protein concentration: An atomic force microscopy study

**DOI:** 10.1002/jemt.23075

**Published:** 2018-10-08

**Authors:** Alberto Moreno‐Cencerrado, Jagoba Iturri, José L. Toca‐Herrera

**Affiliations:** ^1^ Institute for Biophysics, Dept. of Nanobiotechnology, BOKU University for Natural Resources and Life Sciences Muthgasse 11 (Simon Zeisel Haus), Vienna A‐1190 Austria

**Keywords:** Avrami equation, crystal growth, high‐resolution atomic force microscopy, protein adsorption, protein‐substrate interaction

## Abstract

The interplay between protein concentration and (observation) time has been investigated for the adsorption and crystal growth of the bacterial SbpA proteins on hydrophobic fluoride‐functionalized SiO_2_ surfaces. For this purpose, atomic force microscopy (AFM) has been performed in real‐time for monitoring protein crystal growth at different protein concentrations. Results reveal that (1) crystal formation occurs at concentrations above 0.08 µM and (2) the compliance of the formed crystal decreases by increasing protein concentration. All the crystal domains observed presented similar lattice parameters (being the mean value for the unit cell: *a* = 14.8 ± 0.5 nm, *b* = 14.7 ± 0.5 nm, *γ* = 90 ° ± 2). Protein film formation is shown to take place from initial nucleation points which originate a gradual and fast extension of the crystalline domains. The Avrami equation describes well the experimental results. Overall, the results suggest that protein‐substrate interactions prevail over protein–protein interactions.

**Research Highlights:**

- AFM enables to monitor protein crystallization in real‐time.
- AFM high‐resolution determines lattice parameters and viscoelastic properties.
- S‐layer crystal growth rate increases with protein concentration.
- Avrami equation models protein crystal growth.

## 1 INTRODUCTION

Crystal formation, either in nature or in the laboratory, is denoted *crystal growth*. Classically, a crystal is a three dimensional periodic arrangement of atoms (molecules, ions, etc.) in solids, being the texture of the crystals hardly perfect (Kittel, 1996). Investigation of the crystal growth mechanism is crucial for the understanding of some important phenomena such as biomineralization, fractal growth, molecular diffusion, and adsorption (Busch et al., 1999; Clark et al., 2015; Jiang et al., 2017; Loste, Park, Warren, & Meldrum, 2004). Several crystal growth models have been proposed up to date to explain such phenomena. Thus, depending on the parameters that rule the type of crystallization, the theoretical approaches can be classified into two general cases: those depending on the thermodynamic conditions and those led by kinetics (Meldrum & Cölfen, 2008; Rabe, Verdes, & Seeger, 2011; Xu, Ma, & Cölfen, 2007).

Obtaining a controlled *in‐situ* crystal growth in the laboratory represents an experimental challenge. In our particular case, we have chosen as crystallization model system a bacterial protein that is able to form crystalline nano‐arrays in a quite controlled manner. Such protein, SbpA from *Lysinibacillus sphaericus CCM2177*, has the ability to diffuse from solution toward a surface and to subsequently self‐assemble with other neighboring proteins to form a characteristic square (p4) lattice symmetry (Sleytr, Sára, Pum, & Schuster, 2001). This process can be considered merely a kinetic process. Former studies reported that SbpA proteins follow a two‐stage nucleation process (Chung, Shin, Bertozzi, & De Yoreo, 2010; Shin et al., 2012), where the protein‐substrate and protein–protein interactions may play an important role on the pathway toward assembly (Eleta‐López, Moreno‐Flores, Pum, Sleytr, & Toca‐Herrera, 2010). In addition, other studies have revealed that hydrophobic surfaces favor SbpA adsorption kinetics (Eleta‐López, Pum, Sleytr, & Toca‐Herrera, 2011). Among the existing theoretical framework, the Langmuir model (and its derivatives) is known for providing a simple view of the adsorption phenomena occurring in a homogeneous and isotropic fashion along the surface (Rabe et al., 2011). Complementarily, in the particular case of crystal growth, a model that assumes crystal nucleation taking place both homogeneously and isotropically is the Avrami model (Avrami, 1939, 1940, 1941). Joint use of these classical models enables an improved characterization of the system under analysis.

Atomic force microscopy (AFM) is a suitable experimental technique to study protein adsorption, protein crystal growth, and polymer dynamics under controlled thermodynamic conditions, which constitutes a key factor for the study of biological systems in comparison with other high‐resolution techniques (Richter, Him, Tessier, Tessier, & Brisson, 2005). In addition, AFM delivers nanometric resolution on topography and is able to detect forces in the nano\pico Newton range.

In this work, the interplay between protein concentration and (observation) time was investigated for the adsorption and crystal growth of SbpA proteins on fluoride‐functionalized hydrophobic SiO_2_ surfaces, known for their good performance in that sense. The analysis was performed by means of AFM measurements over a range of four protein concentrations (0.08, 0.2, 0.4, and 0.8 µM), which were left to evolve until process completion. The experimental results were then fitted with classical adsorption and crystal growth models which contributed to reinforce the validity of the theoretical approaches defined. These experiments provide new insights into the understanding of protein adsorption and 2D protein crystallization.

## 2 EXPERIMENTAL

### 2.1 Materials

Bacterial surface layer protein SbpA was isolated and purified from *Lysinibacillus sphaericus CCM2177* following the standard protocols (Sleytr, Sára, Küpcü, & Messner, 1986). This protein has a molecular mass of 132 kDa and an isoelectric point of 4.69 (Ilk et al., 2002). Protein recrystallization buffer was prepared with 0.5 mM Trizma base (Sigma, Germany) and 10 mM CaCl_2_ (98% Sigma‐Aldrich, Germany) and adjusted to pH 8. Milli‐Q water (Elga Lab Water Systems, Germany) was used for the preparation of all the solutions employed.

After isolation, the protein solution was centrifuged at 5,000 rpm for 5 min to separate the S‐protein monomers from self‐assembly products and then stored at 4 °C as a 1 mg/ml solution in water. Then, before each experiment, such supernatant solution was diluted using the appropriate amount of recrystallization buffer down to the different corresponding protein concentrations: 0.08 µM, 0.2 µM, 0.4 µM, and 0.8 µM.

### 2.2 Methods

#### 2.2.1 Hydrophobic functionalization

SiO_2_ substrates were chosen as host surface for protein adsorption and crystallization. AFM silicon wafers (IMEC, Belgium) were sonicated in SDS solution, thoroughly rinsed with Milli‐Q water, dried with nitrogen, activated by UV/Ozone treatment and placed overnight into a desiccator under 1H,1H,2H,2H perfluorododecyltricholosilane (Sigma Aldrich, Germany) atmosphere. This procedure turned the SiO_2_ substrates into hydrophobic surfaces with a contact angle value of Θ = 95 ± 5 (see contact angle subsection). Before each experiment, the functionalized SiO_2_ substrates were rinsed again with ethanol and Milli‐Q water, dried under nitrogen and immediately inserted into the fluid chamber of the AFM (see Atomic Force Microscopy subsection). The wettability of the functionalized substrates (measured by contact angle) and their corresponding AFM height micrographs can be found in the Supporting Information Figure S1 of the supporting information.

#### 2.2.2 Contact angle

Measurements were performed with a Kruess Drop Shape Analysis System (Kruess D100, Germany) to confirm differences on surface wettability. Milli‐Q water drops (5 µL) were used as liquid phase. The shapes of the drops were collected by an optical camera and further processed with the software of the equipment. In particular, we have used three different fittings for our calculations: circular, sessile and height/width methods.

#### 2.2.3 Atomic force microscopy

AFM experiments were performed with a Multimode‐AFM (Bruker AXS, USA) controlled by a Nanoscope V and equipped with a JV‐scanner. A fluid chamber was used for performing real‐time measurements. This system was sealed by a silicone O‐ring. Silicon‐nitride probes (DNP‐S, Bruker, USA) were employed in the experiments and their spring constant was calibrated in‐situ (0.3 N/m) at the beginning of each session by means of thermal tune method implemented in the AFM software. Prior to their use in the AFM fluid chamber, the cantilevers were cleaned with UV/Ozone for 20 min. The fluid chamber, tubing, and O‐rings were washed overnight with 2% SDS, rinsed gently with Milli‐Q water, and dried with nitrogen. The AFM fluid chamber offers a volume of 35 ± 5 µL and presents a radius of 0.40 ± 0.05 cm. Therefore, the active area of the surface where the protein crystal can grow is around 0.5 cm^2^.

Once mounted, the system was filled with recrystallization buffer and kept until stabilization of the deflection signal. Then, SbpA proteins were injected into the fluid chamber. AFM images were obtained in tapping mode, at low forces to prevent sample damage and at scan rates lower than 2 Hz. The proportional and integral (PI) gains were individually optimized for each set of experiments. First, the integral gain was increased until the appearance of signal noise. Then, the integral gain was set to its half‐value. Second, the limit of the proportional gain was tested. Finally, the proportional gain was set as a 20% higher than the integral gain. In addition, the oscillation of the cantilever was adjusted to 9.36 kHz and 8 nm in frequency and amplitude respectively.

Thus, three different AFM channels were analyzed: height, amplitude and phase. Whereas height and amplitude describe the topography of the sample under study, phase tapping delivers, in our case, information about the plasticity of the protein layer. This is due to the changes in the phase of the cantilever oscillation while measuring (García & Pérez, 2002). All measurements presented in this work were performed at the same frequency and amplitude values. Therefore, differences in AFM phase imaging within the samples can be related to differences in their viscoelastic properties. A better sample resolution of the AFM phase imaging corresponds to a higher sample stiffness. All the AFM images were processed with the Nanoscope program (Bruker, USA).

#### 2.2.4 Protein crystal area quantification

ImageJ software (National Institutes of Health, USA) was used to calculate the growth of protein crystal layers for each concentration employed. After transforming AFM micrographs to binary images, a threshold between the different gray values was calculated. Then, this color threshold was used to estimate the area of the crystalline protein layer. The program offers different methods based on five different mathematical algorithms. In this manuscript, the results of the image analysis are reported as the mean value of each algorithm type: IsoData, Huang, Li, Mean and Otsu methods (Huang & Wang, 1994; Sezgin & Sankur, 2004; Tajima & Kato, 2011). In addition, the size of each protein crystal domain was calculated with the Nanoscope program.

#### 2.2.5 Data analysis: Avrami equation as crystal growth model

The crystalline area calculated by means of image processing was used for testing applicability of classical crystal growth models. In particular, the Avrami equation (known as Johnson‐Mehl‐Avrami‐Kolmogorov, or JMAK equation) was used to fit the experimental data. The Avrami equation provides the transformed area (A) as a function of time (t):

(1) $$A\left(t\right)=1-e^{-Kt^{n}}$$

where *n* is a constant known as the Avrami exponent (Starink, 2001). This constant presents some restrictions depending on the experimental conditions:

(2) $$n=B+g*N_{\text{dim}}$$

- *B* = 0 if no nucleation occurs during crystal growth, and *B = * 1 if there is constant nucleation at constant rate during crystal growth.
- *g = * 1 for linear growth, and *g = * 1/2 for parabolic growth (diffusion growth).
- *N*_dim_ is equal to the dimensionality of the crystal growth (*N*_dim_* = 1* for 1D crystal growth, *N*_dim_* = 2* for 2D, *and* *N*_dim_  = *3* for 3D).

In this work, we fitted our data with the Avrami equation for values of *n* equal to 1/2, 1, and 2. Among those fittings, the lowest *chi‐square* results (taken as validity factor) corresponded to *n = 1*. The *chi‐square* value is a statistical method to test the goodness of a mathematical fit (Pearson, 1900). Therefore, the lowest *chi‐square* value corresponds to the best fitting of the data.

More specifically, the Equation 3 for the case of SbpA protein crystallization took the following values (confirmed by the statistical analysis):

- *n* = *1*
- *B = 0* (no nucleation occurs during crystal growth)
- *g = * 1/2 (diffusion growth)
- *N*_dim_ *= 2* (two‐dimensional crystallization)

Finally, the second constant in the Avrami equation is *K*. This constant does not have a clear physical meaning and its interpretation depends on the experimental conditions (Jena & Chaturvedi, 1992). In our case, *K* represents a decay rate and its value was calculated as a result of the fitting of the data.

## 3 RESULTS

### 3.1 Crystal structure of the adsorbed protein layer

**Figure 1:**
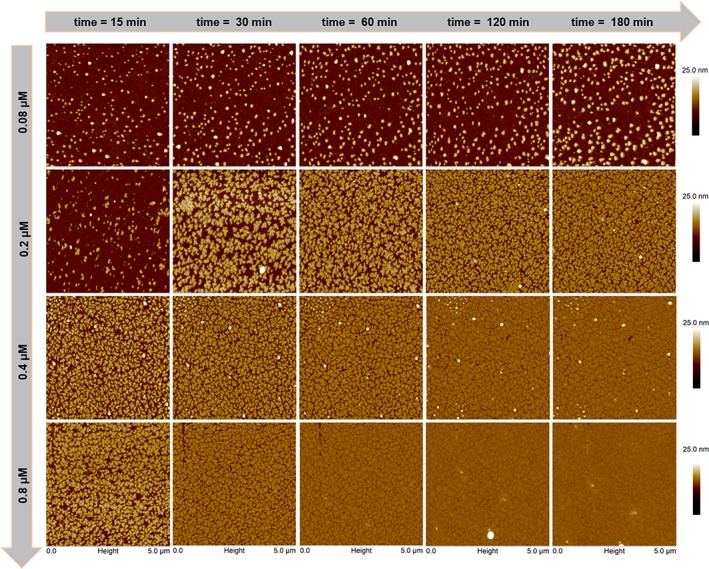
Time‐concentration interplay for crystal growth. A clear increment of the film formation capability can be observed from top‐left to bottom‐right micrographs, as both the SbpA concentration and observation time increase. (Note for the reader: some artifacts, identified with white color, appear and disappear during AFM scanning, but they do not belong to the protein crystal layer) [Color figure can be viewed at wileyonlinelibrary.com]

Figure 1 illustrates the crystal growth in real‐time of SbpA proteins on hydrophobic SiO_2_ surfaces as a function of the protein concentration. The scans were performed for each concentration at five different time sequences after the injection of proteins into the AFM fluid‐chamber: 15, 30, 60, 120, and 180 min. The AFM results show how the interplay of time and concentration influences 2D crystal formation. Thus, the number of nucleation points as well as the size of protein domains increased upon rising both protein concentration and observation time. For the lowest concentration (0.08 µM), the protein domains grew slow and were not able to create a confluent protein layer after the measured observation time. On the contrary, for the highest concentration (0.8 µM), the protein domains grew much faster and the substrate surface was completely covered at 180 min after protein injection. The topography of the functionalized SiO_2_ surfaces was performed as a control experiment and the corresponding AFM height micrographs are plotted in Supporting Information Figure S1 of the Supporting Information.

**Figure 2:**
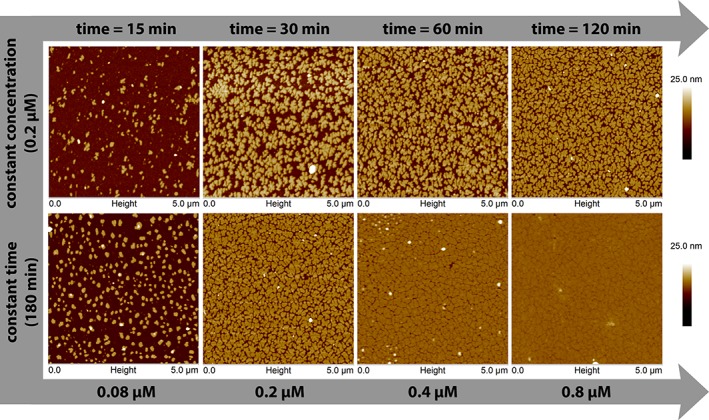
Detailed view of the influence of protein concentration and observation time. Upper row: *in‐situ* AFM height micrographs for a protein concentration of 0.2 µM. After 30 min, protein crystals covered 50% of the surface. 90 min later, 61% of the initial surface was occupied due to crystal growth. Bottom row: influence of the (initial) concentration on the protein crystal layer after 180 min. The smallest concentration was able to occupy the 25% of the initial available surface forming little nonconfluent crystal domains. The coverage and the confluence increased with protein concentration, reaching a maximum value for 0.8 µM [Color figure can be viewed at wileyonlinelibrary.com]

Figure 2 highlights the influence of varying each respective parameter (concentration or time) on both protein adsorption and protein crystal formation. Two particular cases from Figure 1 are shown in detail: (i) one protein concentration (0.2 µM) at different observation times and ii) four protein concentrations after 180 min. The choice of 0.2 µM is explained by the intrinsic kinetics of the crystal growth since higher amounts of SbpA led to an almost immediately coverage of the substrate, as shown in Figure 1. This could have limited the capability to visualize the ongoing evolution of the crystalline film as it grows.

Therefore, on one hand, Figure 2 shows the role of time on crystal formation at one protein concentration. Thus, after 15 min some nucleation points were observed. Profile analysis of these structures revealed that such protein islands had a thickness of about 9 nm. After 30 min, the protein film covered 50% of the available surface. After 60 min, the crystallization process covered 58% of the initial available surface, while at 120 min 61% of the initial surface was covered. Finally, after 180 min the process ended with a surface coverage of 66%. On the other hand, the influence of protein concentration on the final structure obtained was monitored after 180 min, as depicted at the bottom row of Figure 2. Hence, the lowest concentration did not form a complete layer, but only protein islands (thickness = 9 nm approximately). On the contrary, the other concentrations built a confluent layer which grew gradually. The most completed, closed, and homogenous protein layer was obtained for the largest concentration (0.8 µM). The calculated coverage area for every concentration after 180 min is shown in Table 1.

**Table 1:** Obtained data of crystal coverage, size of crystal domains, and lattice parameters as a function of protein concentration after 180 min observation time

Molarity (µM)	Crystal area (%)	Domain diameter (nm)	Lattice parameters	
			*a, b* (nm)	*γ* (°)
**0.8**	90 ± 6	≥ 206	*a* = 15.3 ± 0.3 *b* = 14.9 ± 0.4	90 ± 6
**0.4**	73 ± 4	206 ± 101	*a* = 15.2 ± 0.2 *b* = 15.4 ± 0.3	89 ± 2
**0.2**	66 ± 5	185 ± 70	*a* = 14.9 ± 0.5 *b* = 15.0 ± 0.2	89 ± 2
**0.08**	19 ± 3	122 ± 31	*a* = 13.6 ± 1.0 *b* = 13.6 ± 1.0	89 ± 2

### 3.2 Physical properties of the crystalline protein layer

**Figure 3:**
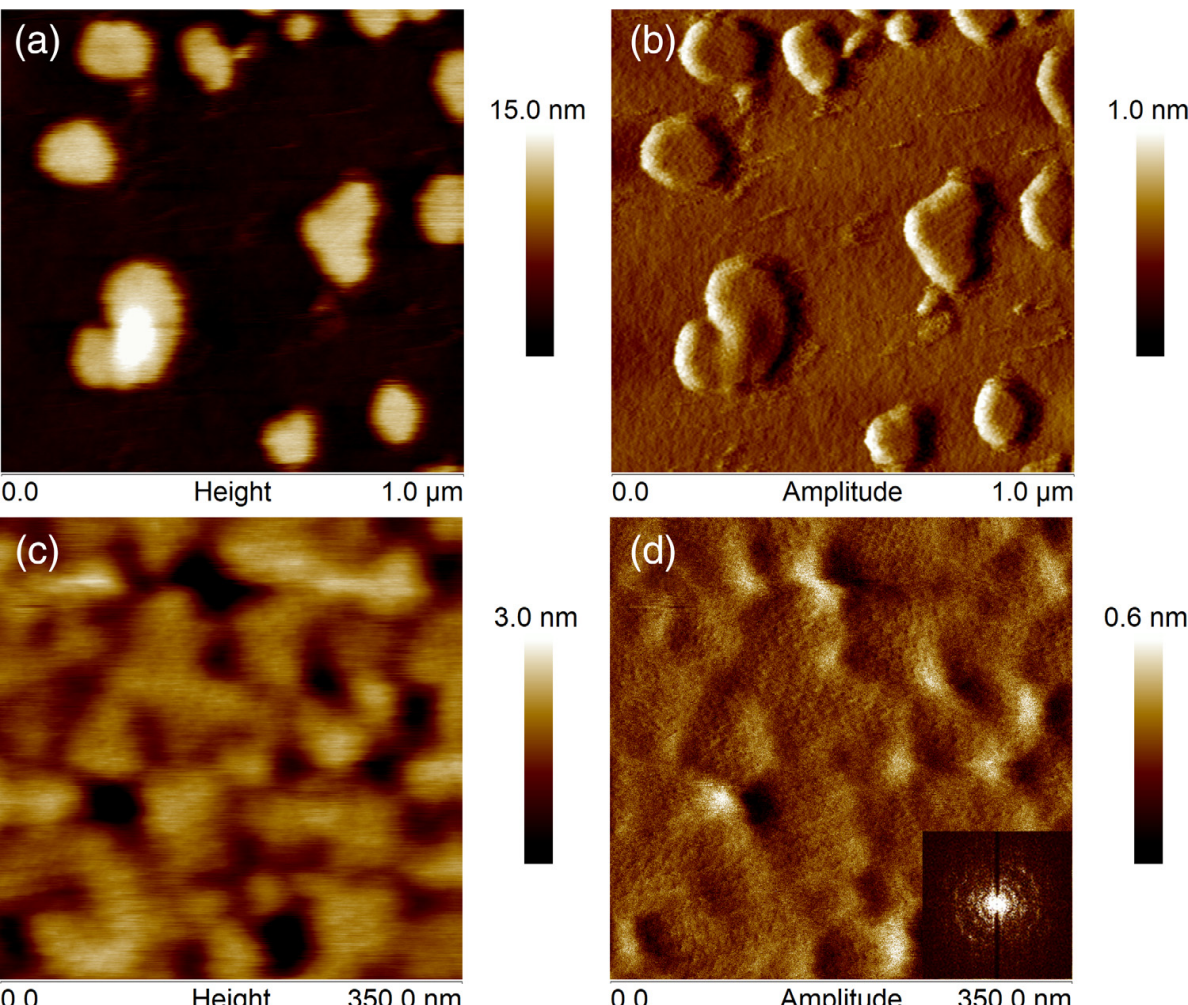
AFM measurements for 0.08 and 0.2 µM protein concentration after 30 min. (a) and (c) AFM micrographs correspond to the protein layer topography at 0.08 and 0.2 µM, respectively. Differently, AFM amplitude images at B) for 0.08 µM and D) for 0.2 µM reveal the existence of crystalline structure already at 30 min. Bottom‐right corner: Fast Fourier Transform image shows the characteristic halo produced by the same lattice at different orientations (lattice parameters are shown in Table 1) [Color figure can be viewed at wileyonlinelibrary.com]

After studying the crystal formation at the micro‐scale, the next step was to examine in more detail the properties of the protein layer at the nano‐scale. This allowed the quantification of the crystal structure and the qualitative description of the viscoelastic properties. Thus, the crystalline lattice of the protein could be visualized at the early steps of the protein crystal growth. Figure 3 illustrates the structure of the protein crystal after 30 min for concentrations of 0.08 µM and 0.2 µM (see AFM images A)‐B) and C)‐D), respectively). In the case of the lowest concentration, the softness of the sample compromised the resolution of the image, being the AFM image somehow distorted by the sample noise (Figure 3A). The increase in resolution of the AFM scans did not delivered better results (Figure 3B). However, the crystalline lattice of the protein could already be distinguished on the error channel of the AFM micrographs at each protein domain (Figure 3B). In the case of 0.2 µM protein concentration, the protein layer seemed to be more rigid and the sample presented much less noise in the image obtained by AFM (see Figure 3C,D). High‐resolution scans confirmed the presence of the basic crystalline structure (Figure 3D) with the following lattice parameters: *a* = 14.9 ± 0.5 nm, *b* = 15.0 ± 0.2 nm, and *γ* = 89 ± 2 °.

**Figure 4:**
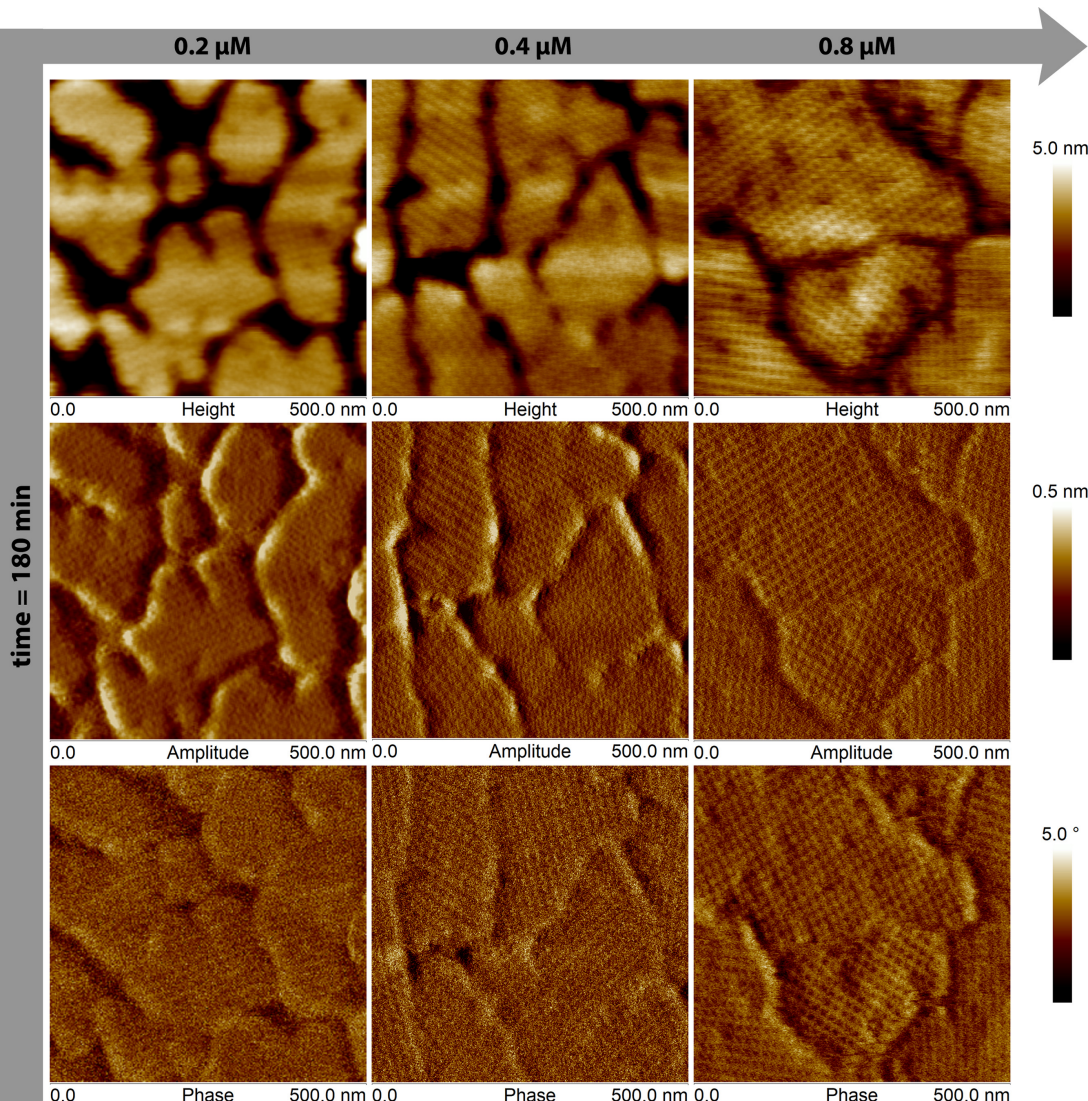
Crystalline protein patterns measured by AFM. Upper row: Topography of the protein layers for each concentration. The coverage of the surface increased with protein concentration. Middle row: Amplitude micrographs, also known as “error” image. The crystalline structure of the protein patterns was clearly solved. All patterns exhibited similar lattice parameters. The bright spots at 0.4 µM and 0.2 µM represent the hits of the AFM tip while measuring. These image artifacts appear when the AFM tip finds a large difference in height. Bottom row: AFM phase images. The protein layer became harder while increasing protein concentration [Color figure can be viewed at wileyonlinelibrary.com]

**Figure 5:**
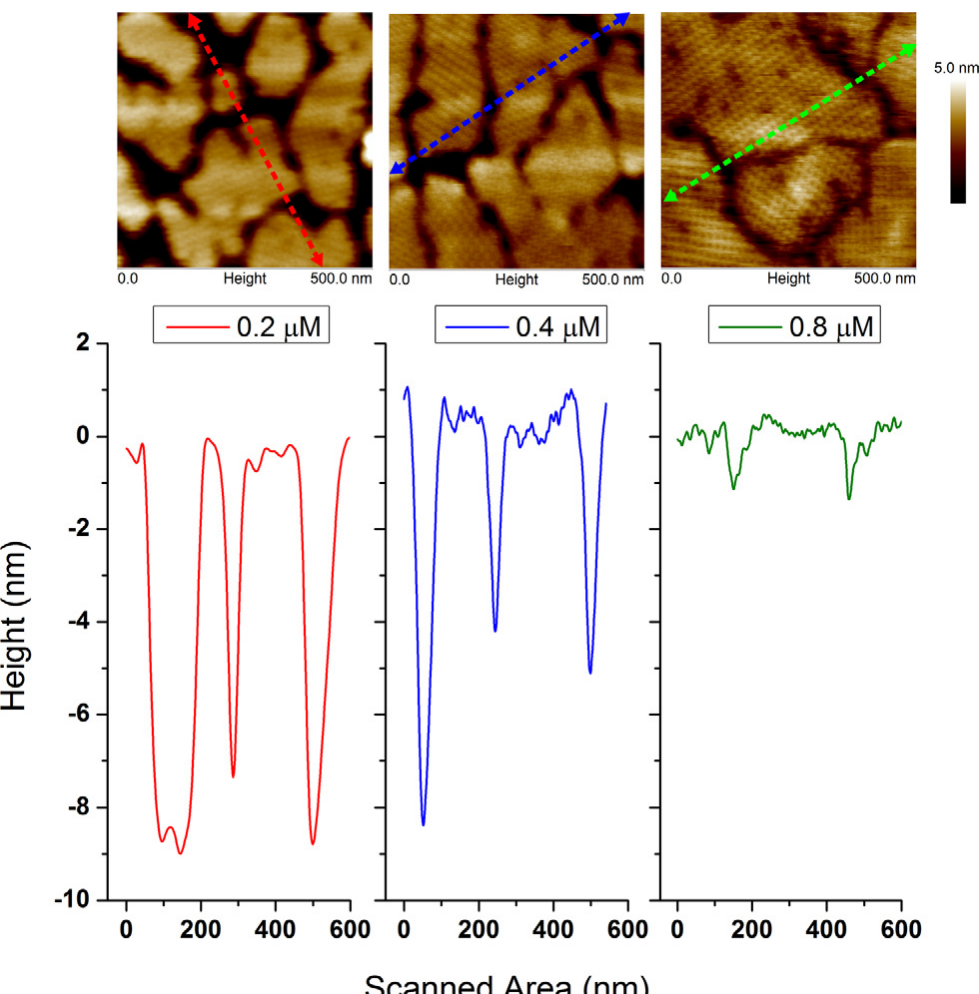
Cross‐section of AFM height micrographs for three different protein concentrations. The analysis is performed across the color arrows. Below: Derived topography profiles from the measurements. Note that for the largest concentration (0.8 µM) the AFM tip does not penetrate between protein crystal domains [Color figure can be viewed at wileyonlinelibrary.com]

The lattice of the protein crystals was also investigated and compared for the three concentrations that formed a confluent protein layer (0.2 µM, 0.4 µM, and 0.8 µM). Thus, high‐resolution AFM scans were performed after 180 min of the protein injection. The results are presented in Figure 4. The upper row shows the AFM height micrographs of the protein crystals. As previously mentioned, the protein layer was more confluent at higher concentrations. A detailed analysis of the crystalline pattern for the three cases revealed similar lattice parameters. These values were calculated with Fourier Fast Transform using the AFM amplitude images, also called AFM error images (see middle row). The viscoelastic behavior of the layer can be obtained from the phase image. The measurements indicate that the protein layer gets harder when increasing the starting protein concentration. In addition, the degree of confluence could be obtained from the AFM height micrographs. Topography cross‐sections performed along each image confirmed that for the highest concentration the protein domains were completely connected to each other. Hence, the height profile given by the AFM tip resulted to be about of 2 nm. On the contrary, at the lowest concentration (0.2 µM), the AFM tip was able to reach the substrate surface delivering a protein layer thickness of about 9 nm. These results are plotted in Figure 5.

## 4 DISCUSSION

The aim of this work was to study the interplay between protein concentration and observation time in 2D crystal growth. For this purpose, we chose bacterial surface layer SbpA as model system. Former studies concerning SbpA have demonstrated that this protein forms p4 symmetric structures on different surfaces such as self‐assembled monolayers (SAMs) (Eleta‐López et al., 2011), polymer brushes (Moreno‐Cencerrado, Iturri, Pum, Sleytr, & Toca‐Herrera, 2016), lipids (Chung et al., 2010), mica (Martin‐Molina et al., 2006), polyelectrolyte multilayers (PEMs) (Delcea et al., 2008), silicon wafers and biopolymers (Toca‐Herrera, Moreno‐Flores, Friedmann, Pum, & Sleytr, 2004). Among them, we have chosen fluoride‐modified hydrophobic silicon dioxide as a host surface for the present study. Previous quartz crystal microbalance (QCM‐D) results (Gołębiewska, 2016) have shown that the affinity of SbpA (estimated with the Langmuir‐Freundlich model) is larger for hydrophobic substrates than for hydrophilic ones (see Supporting Information Figure S2).

The crystal growth can be monitored *in‐situ* by AFM. Among the available AFM scanning modes, tapping mode is suitable to obtain high‐resolution and viscoelasticity information of soft materials. Thus, our results revealed that the formation of a confluent protein crystal layer depends on the initial protein concentration. For instance, for a concentration of 0.08 µM, AFM showed that such layer was not always complete after 180 min (see Figure 1). This does not mean that the self‐assembly of SbpA proteins did not form a 2D protein crystal. In fact, a close look to each round‐shape domain (diameter = 122 nm) exhibited the lattice parameters corresponding to those 2D crystalline layers usually found in bacteria (see Figure 3).

Three more cases were studied and revealed that crystal domains grew more rapidly when increasing the protein concentration. The influence of the interplay of concentration and time on crystal growth is summarized in Figure 2. For a given concentration (0.2 µM), the first nucleation points were created already after 15 min, and protein layer crystal grew exponentially for about 30 min. After this time, the rate of crystal growth became slower because of the lower number of available free adsorption sites for those proteins still diffusing toward the substrate. Figure 2 also provides the information of the total coverage area of the protein crystal after 180 min as a function of protein concentration. As expected, a higher protein concentration produced a more complete and compact crystal layer.

The percentage of the initial surface covered by the protein crystal layer was calculated by ImageJ software (values are shown in Table 1). This type of measurement permitted to determine the degree of incompleteness as well as the defects of the crystal, which should imply different mechanical properties of the protein layer. A visual way to present the role played by both parameters (time and concentration) is to build a (4 × 5) matrix, where each element denotes the percentage of the area covered by the protein crystal domains:

$$\left(\begin{array}{cc} \begin{array}{ccc} a_{11} & a_{12} & a_{13} \end{array} & \begin{array}{cc} a_{14} & a_{15} \end{array}\\ \begin{array}{ccc} \begin{array}{c} a_{21}\\ a_{31}\\ a_{41} \end{array} & \begin{array}{c} a_{22}\\ a_{32}\\ a_{42} \end{array} & \begin{array}{c} a_{23}\\ a_{33}\\ a_{43} \end{array} \end{array} & \begin{array}{cc} \begin{array}{c} a_{24}\\ a_{34}\\ a_{44} \end{array} & \begin{array}{c} a_{25}\\ a_{35}\\ a_{45} \end{array} \end{array} \end{array}\right)=A_{ij}\to \{\begin{array}{c} a_{ij}=\%\text{covered area}\\ i=\text{protein concentration}\\ j=\text{observation time} \end{array}$$

Note that for a constant protein concentration value (e.g., *i* = 1) the observation time increases from 1 to 5, and vice versa. Thus, the surface coverage values (mean value ± standard deviation) where calculated from the AFM images of Figure 1 with ImageJ (see Experimental Section).

$$A_{ij}=\left(\begin{array}{cc} \begin{array}{ccc} \;8\pm 2 & \;15\pm 1 & 15\;\pm 1 \end{array} & \begin{array}{cc} 16\pm 2 & 19\pm 3 \end{array}\\ \begin{array}{ccc} \begin{array}{c} 15\pm 2\\ 50\pm 3\\ 58\pm 4 \end{array} & \begin{array}{c} 50\pm 4\\ 58\pm 5\\ 61\pm 6 \end{array} & \begin{array}{c} 58\pm 4\\ 64\pm 5\\ 73\pm 5 \end{array} \end{array} & \begin{array}{cc} \begin{array}{c} 61\pm 5\\ 73\pm 6\\ 89\pm 6 \end{array} & \begin{array}{c} 66\pm 5\\ 73\pm 6\\ 90\pm 6 \end{array} \end{array} \end{array}\right)$$

For simplicity, the matrix is rewritten without the standard deviation. The last column in the matrix (covered area at 180 min) delivers almost the same percentage as the previous column (measured after 120 min) and therefore can be neglected:

$$A_{ij}=\left(\begin{array}{cc} \begin{array}{ccc} 8 & 15 & 15 \end{array} & 16\\ \begin{array}{ccc} \begin{array}{c} 15\\ 50\\ 58 \end{array} & \begin{array}{c} 50\\ 58\\ 61 \end{array} & \begin{array}{c} 58\\ 64\\ 73 \end{array} \end{array} & \begin{array}{c} 61\\ 73\\ 89 \end{array} \end{array}\right)$$

The matrix indicates that there are a number of combinations between concentration and time that delivered the same surface coverage. For example, lower concentrations and longer observation times produce the same covered area as higher concentrations and shorter times. This can be seen by the symmetry of the values (in red) with respect to the diagonal (numbers marked in bold). Such representation could be useful to plan future experiments.

But, is it possible to gain more information about the structure of the protein layer? Does it depend on the protein concentration? According to the Fourier analysis of the experimental results, concentrations larger than 0.08 µM led to almost the same lattice parameters (see Table 1), although the total crystal size increased when the concentration was raised. Figure 4 reveals more information than exclusively the crystalline structure of the formed protein layers. The AFM phase images confirmed that a more compact protein layer was achieved at higher concentrations. This can be explained by taking into account how for higher protein concentrations the surface offers less freely standing binding sites and, at the same time, the gaps between the growing crystalline domains are gradually reduced. The hardest protein layer and, therefore, the layer that dissipates less energy while being tapped by the AFM tip corresponds to a complete (full coverage) protein crystal layer. In addition, former studies already reported that the softness of the samples affects the AFM resolution (Radmacher, Fritz, & Hansma, 1995). Thus, in our results, the best resolution in AFM phase imaging corresponds with the higher protein concentration.

Regarding the thickness of the protein crystal layer, Figure 5 shows different layer thicknesses as a function of the protein concentration. This result can be explained by taking into account the degree of confluence of each sample. The study of the layer thickness for isolated domains (before the crystal growth has finished) revealed the same value for all concentrations (≈9 nm), which is in agreement with previous results obtained for hydrophobic SiO_2_ substrates (Györvary, Stein, Pum, & Sleytr, 2003).

**Figure 6:**
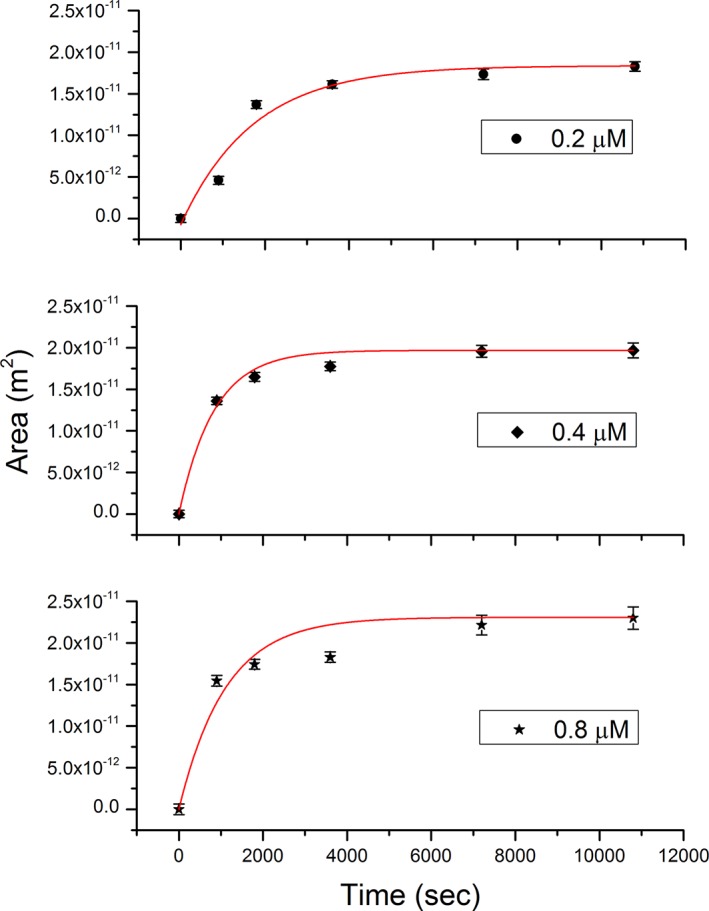
Avrami model for crystal growth at different concentrations. The points in each graph represent the protein crystal area measured by AFM for each concentration. These areas have been calculated with ImageJ software [Color figure can be viewed at wileyonlinelibrary.com]

Protein concentration also influenced the final size of the protein crystal layer. Therefore, it turned out to be quite interesting to quantify such a dependency. Figure 6 shows the area of the protein crystal as a function of time for the following protein concentrations: 0.2 µM, 0.4 µM and 0.8 µM. Among the models used to model crystal growth, we have chosen the Avrami equation due to the simplicity of our system. Other studies reported more complex situations that take into account crystal growth with limited resources also included the Gompertz function to fit the experimental results (Bolós, Benítez, Eleta‐López, & Toca‐Herrera, 2018). The Avrami model can be applied when protein nucleation occurs randomly and homogenously over the whole surface, where the crystal grows at the same rate in all directions without depending on crystal size. The general equation related to the model of Avrami (for more details, see Experimental Section) is the following:

(3) $$Y=A_{1}-A_{2}\text{exp}(-Kt^{n})$$

In our case, ***Y*** represents the total area of the protein crystal, while ***t*** is the observation time. For very large experimental time, *Y = A*_1_ (expressed in m^2^); whereas for time = 0, *A*_1_ has to be equal to *A*_2_. The equation has two other main parameters: ***n*** and ***K***. For 2D growth ***n*** is equal to one (Christian, 2002; Starink, 2001), while ***K*** represents a decay rate. The validity of the fitting of the crystal growth data by this equation was tested by the chi‐square value obtained for the three concentrations.

The calculated rates were: *K*_0.2_ = 5.8E‐4 s^−1^, *K*_0.4_ = 14E‐4 s^−1^, and *K*_0.8_ = 9.2E‐4 s^−1^ for 0.2 µM, 0.4 µM, and 0.8 µM, respectively. These values indicate that the rate of crystal growth is faster for larger concentrations (between two and three fold). The fitting “goodness” indicates that the Avrami equation, which is a 1‐step model, is adequate to describe SbpA crystal growth. This result appears as the first fact to establish a prominence of the protein‐substrate interactions over the protein–protein interactions in the crystal layer formation. Since the Avrami equation assumes as the main parameter the presence and amount of nucleation points, the goodness of the method might imply that the same theoretical mechanism would apply for the SbpA protein crystal growth.

Protein–protein interactions are mainly responsible for the lattice symmetry of the crystal, which remains unalterable for all the concentrations studied in this work. On the contrary, the adsorption kinetics, the number of nucleation points and the further protein crystal growth are parameters related to the surface interactions. All these parameters are affected by the protein concentration. Thus, the protein‐substrate interactions seem to be more sensible upon any change in the experimental conditions, which would also speak about their importance in the crystal layer formation.

At this point, we have to mention the limitations of the experimental procedure due to the fast adsorption kinetics of the SbpA protein on these surfaces. In fact, AFM imaging started after the hydrophobic surface was already exposed to the protein solution for about 15 min, time enough to find the smallest crystalline domains (see Figure 3). Although these first adsorption/recrystallization moments could not be directly visualized by AFM, former QCM‐D results have shown that this process is mainly dominated by protein‐substrate interactions at this time scale, followed by further protein self‐assembly (Eleta‐López et al., 2010, 2011; Moreno‐Cencerrado et al., 2016).

## 5 CONCLUSIONS

AFM represents a robust method to study protein adsorption and two‐dimensional protein crystal growth. In the case of SbpA, concentrations equal or larger than 0.08 µM led to crystalline protein structure. According to the analysis of the results, protein‐substrate interactions dominated over protein–protein interactions in SbpA crystal formation (nucleation and crystal growth). Although the lattice parameters remained invariant, the compliance of the final crystal depended on the initial protein concentration. This fact is directly related to the confluence and the boundary defects of the observed crystal domains.

A remaining open question would be whether larger experimental times would contribute to the confluence of the crystal at lower protein concentrations (above 0.08 µM). This work shows that AFM and digital imaging processes can provide new insights on molecular self‐assembly and crystal growth. Furthermore, the results obtained with such techniques can be used to test and develop theoretical models and theories (i.e., self‐assembly, crystal growth, etc.).

## Supporting information

Supporting Informations
