# Supplementary material for: In‐situ 2D bacterial crystal growth as a function of protein concentration: An atomic force microscopy study
Source: Microsc Res Tech. 2018 Oct 8;81(10):1095–104. doi: 10.1002/jemt.23075 (PMC6704365; doi:10.1002/jemt.23075)
Supplement: Supplementary file 1 — Supporting Informations [file JEMT-81-1095-s001.docx]

**Supporting Information**

***In situ 2D bacterial crystal growth as a function of protein concentration: an atomic force microscopy study***

Alberto Moreno-Cencerrado, Jagoba Iturri, and José L. Toca-Herrera*

Address: Institute for Biophysics, Dept. of Nanobiotechnology, BOKU University for Natural Resources and Life Sciences, Muthgasse 11 (Simon Zeisel Haus), A-1190 Vienna (Austria)

* Corresponding authors

Email: Prof. José L. Toca-Herrera - [jose.toca-herrera@boku.ac.at](mailto:jose.toca-herrera@boku.ac.at)


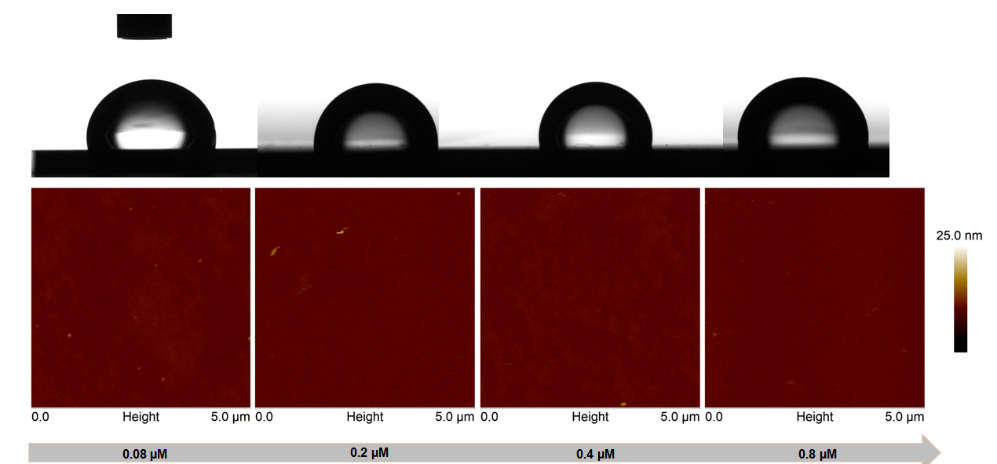


**Figure S-1: Contact angle and AFM height micrographs of the fluorosilane-functionalized SiO_2_ surfaces used for investigating each protein concentration.** All the contact angle measurements delivered values above 90° (95±5° in average). AFM images of the functionalized silicon surface revealed a homogenous, flat, clean surface before exposure to the protein solution. The AFM results show the topography of the fluorosilane-functionalized SiO_2_ surfaces before exposure to the protein concentration.

**
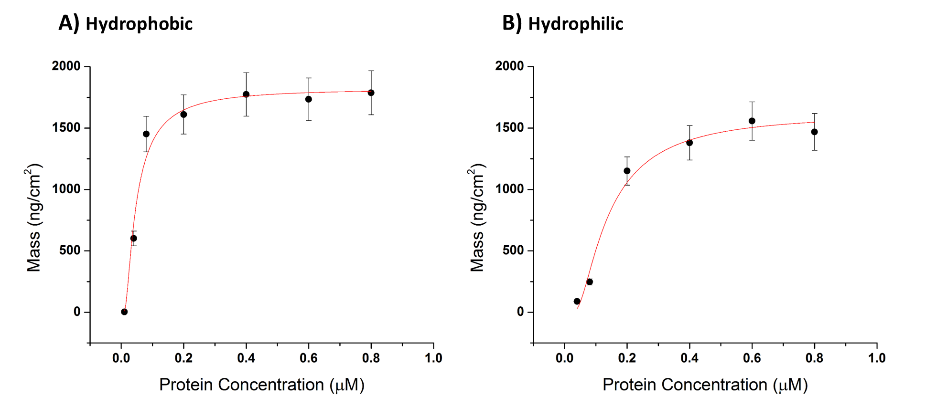
**

**Figure S-2.** Adsorption isotherms as a function of the protein concentrations. The protein affinity seems to be higher for hydrophobic surfaces. This can be attained by measuring the changes in frequency versus protein concentration (isotherms). The experimental points were fitted with the Langmuir-Freundlich equation:

$Q={(Q}_{sat}{Kc^{n})}/{(1+Kc^{n})}$

In this equation, *Q* is the adsorbed protein mass, *Q_sat_* is the adsorption capacity of the system, *c* is the protein concentration, *K* is the adsorption affinity constant and *n* is an indicator of heterogeneity. In the Figure, A) represents a hydrophobic (fluorosilane-coated) SiO_2_ substrate, while B) relates to hydrophilic (UV/Ozone treated) SiO_2_ substrate. In the case of hydrophobic SiO_2_ substrates, this value corresponds to K_phobic_ = 88 ± 25 mg^-1^. In the case if hydrophilic SiO_2_ substrates, this value drops to K_philic_ = 20 ± 3 mg^-1^.

**
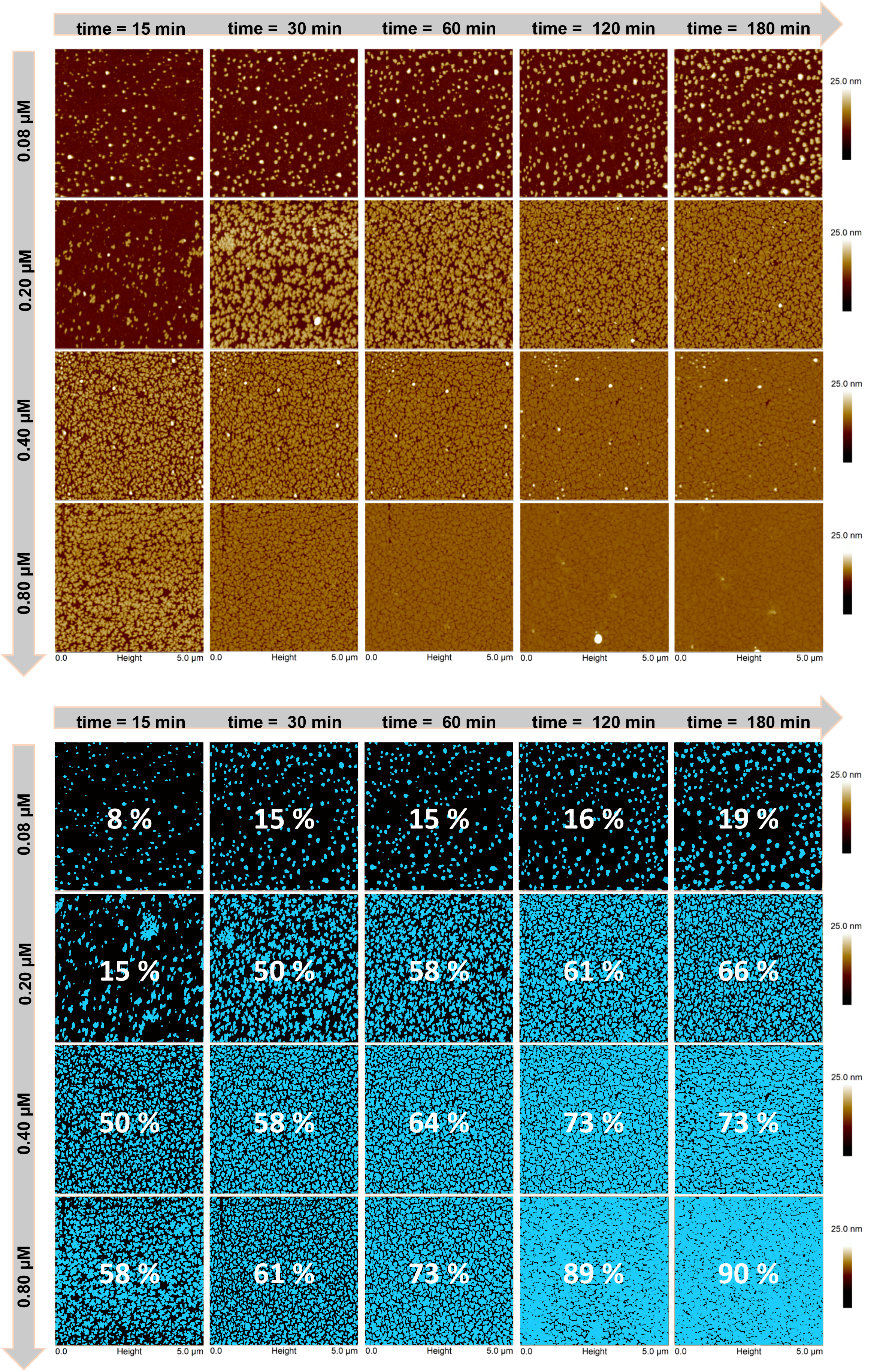
**

**Figure S-3.** Comparison of AFM height images with the surface profiles calculated by ImageJ.

| Time  (minutes) | Area 1: 0.20 µM  (m^2^) | Area 2: 0.40 µM  (m^2^) | Area 3: 0.80 µM  (m^2^) | Percentage coverage:  0.20 µM | Percentage coverage:  0.40 µM | Percentage coverage:  0.80 µM |
| --- | --- | --- | --- | --- | --- | --- |
| 15 | 4.6E-12 | 13.60E-12 | 15.82E-12 | 20% | 59% | 69% |
| 30 | 13.7E-12 | 16.50E-12 | 17.43E-12 | 60% | 71% | 76% |
| 60 | 16.12E-12 | 17.76E-12 | 18.29E-12 | 70% | 77% | 80% |
| 120 | 17.36E-12 | 19.57E-12 | 22.15E-12 | 75% | 85% | 96% |
| 180 | 18.28E-12 | 19.68E-12 | 22.99E-12 | 79% | 86% | 100% |

**Table S-1. Comparison of the protein crystal growth values used in the Avrami equation:** The four first columns show the values of the area of the protein crystal layer as a function of the time for each concentration under analysis. (NOTE: these results are shown in Figure 6 of the manuscript and they were used for the fitting with the equation of Avrami). The last three columns show the normalization of the area (percentage coverage) as a function of the time for the three concentration. All the values have been normalized by the highest coverage area (22.99 E‑12 m^2^).


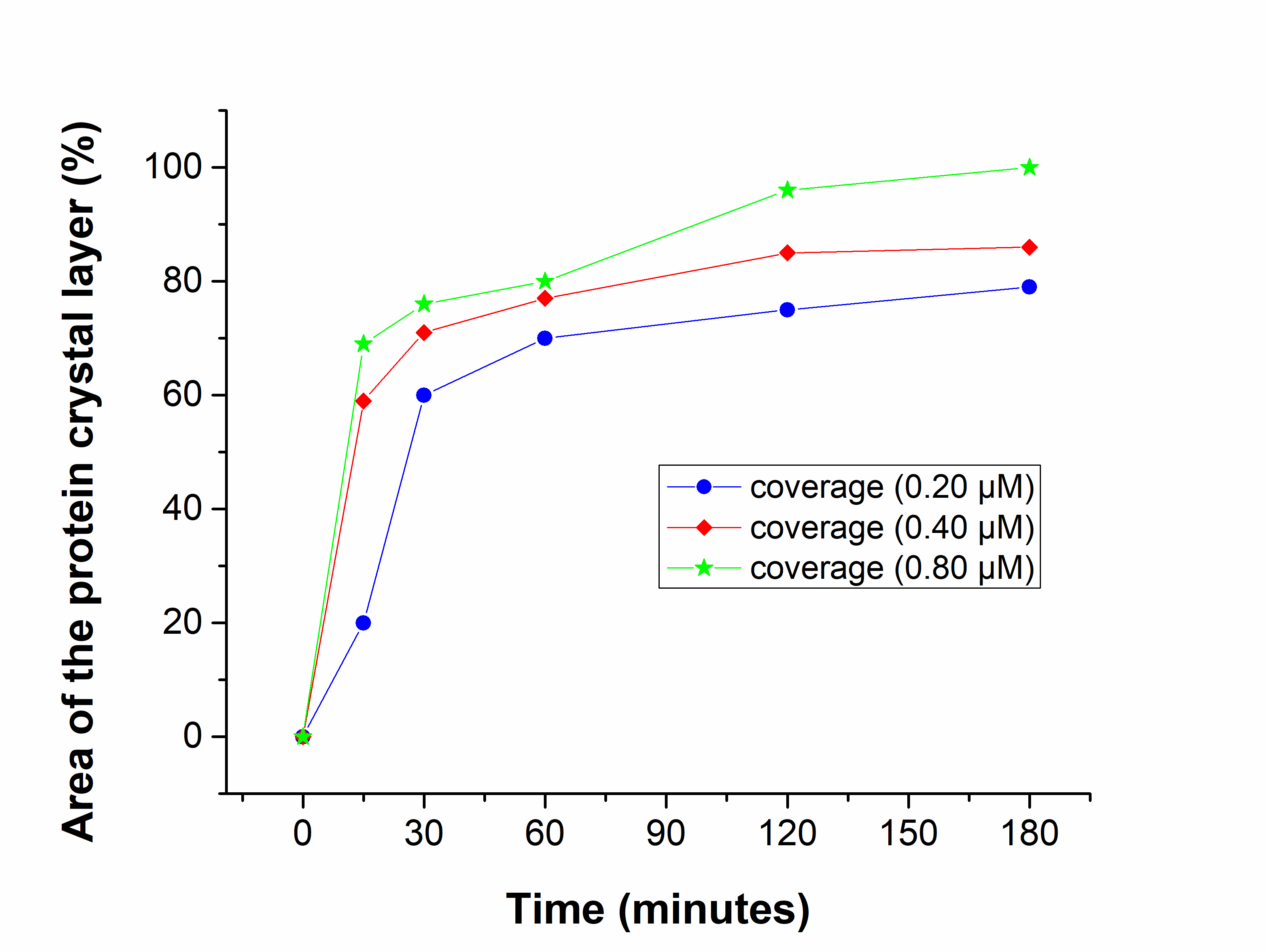


**Figure S-4. Illustration of the results from Table S-1.**
